# Supplementary material for: Physiological response and transcriptome analyses of leguminous Indigofera bungeana Walp. to drought stress
Source: PeerJ. 2023 Jun 14;11:e15440. doi: 10.7717/peerj.15440 (PMC10276564; doi:10.7717/peerj.15440)
Supplement: Supplemental Information 7 [file peerj-11-15440-s007.docx]

| Group | RNASeq Power |
| --- | --- |
| MJWCKL1_MJWCKL2_MJWCKL3_vs_MJWTrL1_MJWTrL2_MJWTrL3 | 0.92 |
| MJWCKR1_MJWCKR2_MJWCKR3_vs_MJWTrR1_MJWTrR2_MJWTrR3 | 0.99 |
